# Targeting the RNA m^6^A modification for cancer immunotherapy

**DOI:** 10.1186/s12943-022-01558-0

**Published:** 2022-03-16

**Authors:** Xinxin Li, Shoubao Ma, Youcai Deng, Ping Yi, Jianhua Yu

**Affiliations:** 1 Xi’an Key Laboratory of Stem Cell and Regenerative Medicine, Institute of Medical Research, Northwestern Polytechnical University, Xi’an, Shaanxi 710072 P. R. China; 2 Department of Hematology and Hematopoietic Cell Transplantation, City of Hope National Medical Center, Los Angeles, CA USA; 3 Institute of Materia Medica, College of Pharmacy, Army Medical University, Chongqing, P. R. China; 4 Department of Obstetrics and Gynecology, The Third Affiliated Hospital of Chongqing Medical University, Chongqing, 401120 P. R. China; 5 Department of Immuno-Oncology, City of Hope Comprehensive Cancer Centre, Beckman Research Institute, Los Angeles, CA USA; 6 Hematologic Malignancies Research Institute, City of Hope National Medical Center, 1500 East Duarte, Los Angeles, CA 91010 USA

**Keywords:** Tumor microenvironment, Cancer immunotherapy, Epigenetics, *N*^6^-methyladenosine, m^6^A modification, m^6^A regulators

## Abstract

*N*^6^-methyladenosine (m^6^A) is the most abundant epigenetic modification of RNA, and its dysregulation drives aberrant transcription and translation programs that promote cancer occurrence and progression. Although defective gene regulation resulting from m^6^A often affects oncogenic and tumor-suppressing networks, m^6^A can also modulate tumor immunogenicity and immune cells involved in anti-tumor responses. Understanding this counterintuitive concept can aid the design of new drugs that target m^6^A to potentially improve the outcomes of cancer immunotherapies. Here, we provide an up-to-date and comprehensive overview of how m^6^A modifications intrinsically affect immune cells and how alterations in tumor cell m^6^A modifications extrinsically affect immune cell responses in the tumor microenvironment (TME). We also review strategies for modulating endogenous anti-tumor immunity and discuss the challenge of reshaping the TME. Strategies include: combining specific and efficient inhibitors against m^6^A regulators with immune checkpoint blockers; generating an effective programmable m^6^A gene-editing system that enables efficient manipulation of individual m^6^A sites; establishing an effective m^6^A modification system to enhance anti-tumor immune responses in T cells or natural killer cells; and using nanoparticles that specifically target tumor-associated macrophages (TAMs) to deliver messenger RNA or small interfering RNA of m^6^A-related molecules that repolarize TAMs, enabling them to remodel the TME. The goal of this review is to help the field understand how m^6^A modifications intrinsically and extrinsically shape immune responses in the TME so that better cancer immunotherapy can be designed and developed.

## Background

Recent studies of RNA modifications have revealed that RNA is not simply an intermediary between DNA and protein or an effector molecule [like ribosomal RNA (rRNA) and transfer RNA (tRNA)] but that it also plays significant roles in post-transcriptional gene regulation [1]. To date, more than 100 types of chemical modification have been identified in cellular RNAs, including *N*^1^-methyladenosine (m^1^A), *N*^6^-methyladenosine (m^6^A), 5-methylcytosine (m^5^C), *N*^7^-methylguanosine (m^7^G), RNA cap methylations, pseudouridine, and uridylation [2]. Among these, m^6^A, which forms when adenosine is methylated at the nitrogen-6 position, is the most abundant and conserved internal RNA modification [1]. More recently, the identification of methyltransferase, demethylase, and binding proteins that install, remove or recognize m^6^A has revealed unappreciated roles of m^6^A in almost every aspect of RNA metabolism as well as in various physiological and pathological processes [3, 4].

Recent studies have revealed that the m^6^A modification modulated immune cell activation and infiltration into the tumor microenvironment (TME) and thus may affect the efficacy of immunotherapy. Therefore, the m^6^A modification is a potential target for cancer immunotherapy that could perhaps complement immune checkpoint inhibitor therapies and chimeric antigen receptor (CAR) T cell therapy, which have dramatically improved the survival and quality of life for cancer patients [5, 6]. Here, we provide an up-to-date and comprehensive overview of the m^6^A modification in immune cells and associated anti-tumor immune responses in the TME. Additionally, we discuss the potential therapeutic value of targeting m^6^A regulators for cancer immunotherapy.

## Regulation of the m^6^A modification

**Fig. 1:**
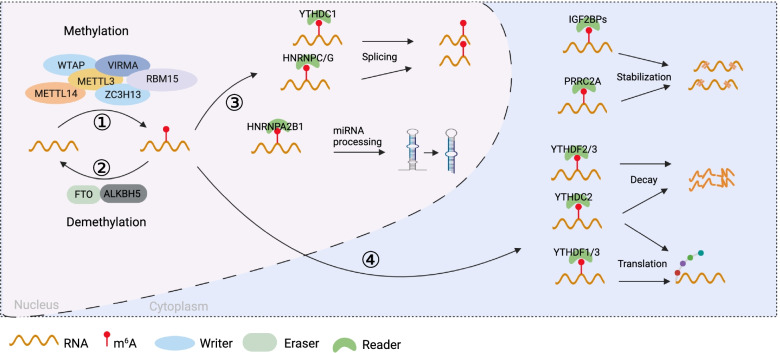
Overview of the m^6^A modification in the mRNA life cycle. ①The m^6^A methylation complex, which consists of the core methyltransferase-like protein 3 (METTL3) and its adaptors (writer), adds m^6^A onto target RNAs in the nucleus. ②The two main demethylases (eraser), fat mass and obesity-associated protein (FTO) and alkB homolog 5, RNA demethylase (ALKBH5), erase the methylation modification in the nucleus. ③ m^6^A is recognized by diverse readers, such as YTHDC1, HNRNPC/G, and HNRNPA2B1, that mediate various posttranscriptional processes including RNA splicing and miRNA processing in the nucleus. ④ In the cytoplasm, m^6^A binds to different specific reader proteins, such as IGF2BPs, PRRC2A, YTHDF1/2/3, and YTHDC2, that mediate the stability, translation, and decay of the mRNA. Figure created with BioRender.com

The RNA m^6^A modification process is dynamically and reversibly regulated by three types of enzymes: m^6^A methyltransferases (“writers”), m^6^A demethylases (“erasers”), and m^6^A binding proteins (“readers”) [7]. Together, these enzymes ensure normal expression and translation of RNAs [8, 9]. The regulation and functions of the m^6^A regulators are summarized in Fig. 1.

Installation of m^6^A is catalyzed by a big writer complex, which consists of methyltransferase-like 3 (METTL3), methyltransferase-like 14 (METTL14), RNA binding motif protein 15 (RBM15), wlms tumor 1 associated protein (WTAP), vir-like m^6^A methyltransferase associated (VIRMA) subunits, and zinc finger CCCH domain-containing protein 13 (ZC3H13) [10]. METTL3 is the predominant catalytic subunit of the writer complex, but its catalytic activity is enhanced by METTL14, an allosteric activator [11, 12]. WTAP, a regulatory component of the complex, recruits the METTL3/METTL14 complex to form a catalytic core on a target RNA [13]. RBM15, an interacting partner of WTAP, is an adapter protein that recruits the m^6^A writer complex to U-rich regions of mRNA [14]. VIRMA, another important subunit of the writer complex, can situate the m^6^A modification in the 3′UTR near stop codon [15]. ZC3H13 controls nuclear m^6^A methylation by combining with other cofactors, such as WTAP and RBM15 [16]. This writer complex usually installs the m^6^A modification on a specific and consensus RNA sequence—RRACH (R = G or A; H = U, A or C)—that is often found in 3′ untranslated regions (UTRs), long exons, and near stop codons [17, 18].

Erasers remove the m^6^A decoration from RNA by removing the adenosine [19]. The two main erasers are fat mass and obesity-associated protein (FTO) and alpha-ketoglutarate-dependent dioxygenase alkB homologue 5 (ALKBH5) [20, 21]. It is now clear that erasers can have different biological effects depending on which specific target RNAs they demethylate. However, what leads to this selectivity is still unknown and needs to be addressed.

A number of different readers recognize the m^6^A decorations of target genes. According to their location, readers can be further divided into nuclear readers and cytoplasmic readers. The nuclear readers, which consist of YTH domain containing 1–2 (YTHDC1, YTHDC2), members of the heterogeneous nuclear ribonuclease (HNRNP) family (HNRNPA2B1, HNRNPC, HNRNPG), and fragile x mental retardation protein (FMRP), play multiple roles in modulating mRNA splicing, epigenetic silencing, nuclear export of mRNA, regulation of non-coding RNA, and RNA structure switching [22–25]. The cytoplasmic readers affect mRNA stability, translation, and degradation, and they include YTHDC2, YTH domain family 1–3 (YTHDF1, YTHDF2, YTHDF3), insulin-like factor-2 mRNA-binding proteins (IGF2BPs), and proline-rich coiled-coil 2A (PRRC2A) [26–28].

## Role of the m^6^A modification in immune cells

It is widely known that intrinsic m^6^A modification regulates tumor cell fate by targeting specific genes in different cancers [29, 30]. However, few studies have focused on how m^6^A modification regulates anti-tumor functions of immune cells. Here, we summarize recent findings.

### Natural killer cells

**Fig. 2:**
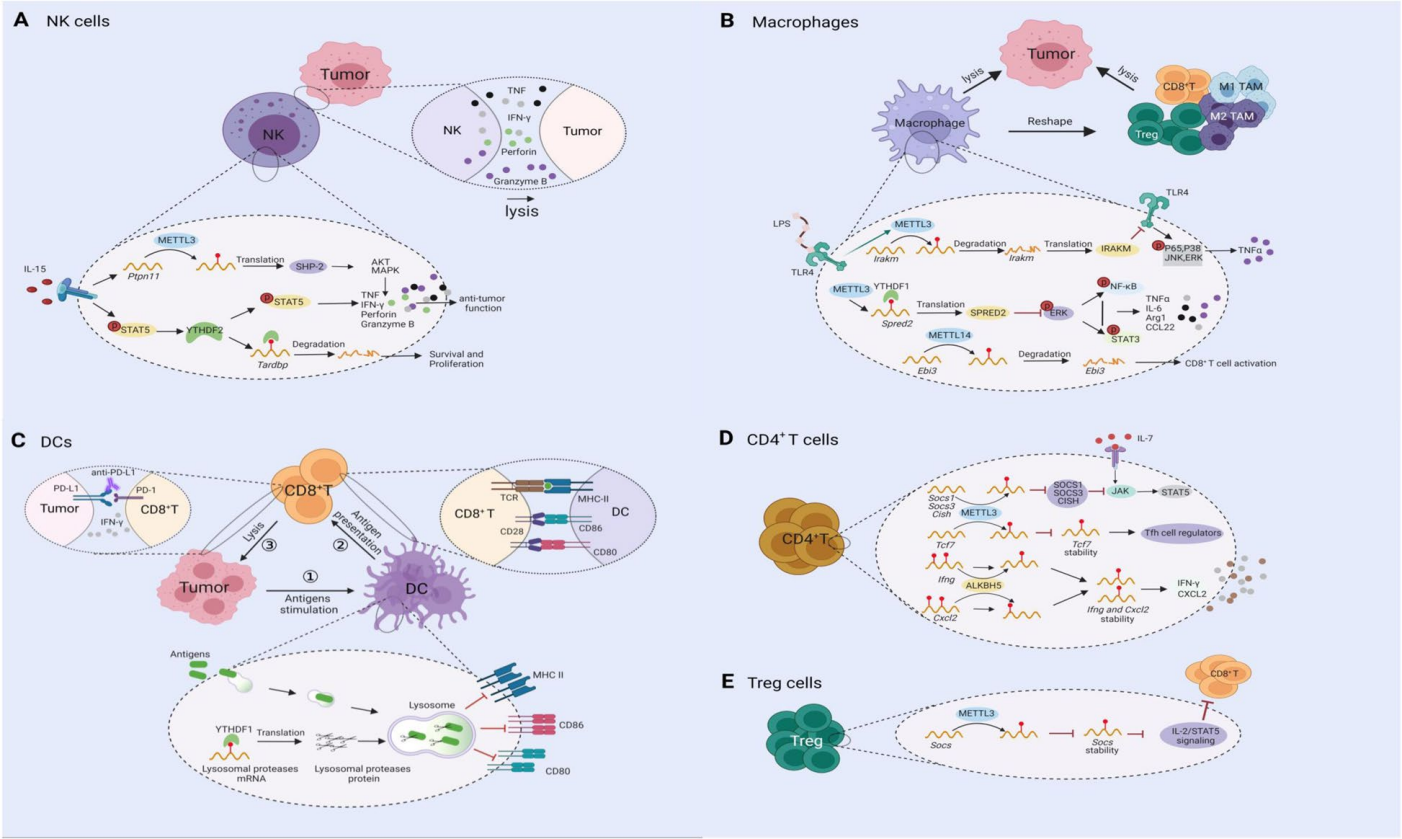
Mechanisms that regulate the m^6^A modification in immune cells. **A** METTL3 (a writer) and YTHDF2 (a reader) positively regulate the survival and anti-tumor immunity of NK cells by respectively targeting *Ptpn11,* STAT5, or *Tardbp.* **B** METTL3 in macrophages promotes the production of proinflammatory cytokines such as TNF-α and IL-6 by targeting *Irakm* (an inhibitor of TLR4) or *Spred2* (an inhibitor of the ERK pathway), thereby reshaping the TME and inhibiting tumor progression. METTL14-mediated m^6^A methylation boosts the degradation of *Ebi3* in macrophages, which promotes CD8^+^ T cell activation and inhibits tumor growth. **C** YTHDF1 in dendritic cells (DCs) enhances the translation of mRNAs encoding proteases that can degrade antigens inside lysosomes. Without YTHDF1, the translation of lysosomal proteases wanes, favoring antigen cross-presentation and promoting more CD8^+^ T cell responses against tumors. **D** In CD4^+^ T cells, METTL3 inhibits the expression of the SOCS family proteins (SOCS1, SOCS3, and CISH), which inhibit JAK, thereby enhancing the activation of IL-7-mediated JAK/STAT5 to ultimately promote the homeostasis and differentiation of CD4^+^ T cells. METTL3 also reduces the stability of *Tcf7* mRNA, promotes the expression of T follicular helper (Tfh) cell regulators, and subsequently enhances the functional maturation of Tfh cells. ALKBH5 decreases m^6^A modification on interferon-γ and C-X-C motif chemokine ligand 2 mRNA, increasing the stability of their mRNAs and boosting the expression of their proteins in CD4^+^ T cells. **E** METTL3 in regulatory T (Treg) cells reduces the stability of *Socs* mRNA by m^6^A modification. This activates IL-2/STAT5 signaling and inhibits the secretion of T cell effector cytokines, diminishing the anti-tumor response of effector T cells, such as CD8^+^ T cells, in the TME. Figure created with BioRender.com

Natural killer (NK) cells are innate lymphoid immune cells with important roles in cancer immune surveillance due to their ability to directly recognize and kill cancer cells [31, 32]. Our group first reported the multifaceted roles of YTHDF2-mediated m^6^A methylation in NK cell immunity [33]. This important m^6^A reader is critical for maintaining NK cell homeostasis, maturation, interleukin (IL)-15-mediated survival, and antitumor and antiviral activity because it regulates several downstream target genes, including signal transducer and activator of transcription 5 (STAT5), Eemesodermin (Eomes), and *Tardbp* [33]. Subsequently, Chen et al. observed that mRNA levels of the m^6^A writer METTL3 were decreased in tumor-infiltrating NK cells of certain cancer patients. Using mouse models, they also showed that deleting METTL3 reduced NK cell hyporesponsiveness to IL-15, promoted tumor progression and metastasis by targeting SH2 domain-containing protein tyrosine phosphatase-2 (SHP-2) [34]. As both the m^6^A writer METTL3 and the reader YTHDF2 positively regulate the anti-tumor immunity of NK cells, METTL3- and YTHDF2-mediated m^6^A methylation might be important regulators of anti-tumor immunity and homeostasis of NK cells (Fig. 2A) [34]. However, the effector functions and regulatory mechanisms of other m^6^A regulators on NK cells remain to be determined.

### Macrophages

Macrophages are phagocytic cells of the innate immune system and mainly involved in the recognition, phagocytosis, and degradation of pathogens and tumor cells [35]. Macrophages are highly involved in tumor initiation and progression. Specifically, tumor-associated macrophages (TAMs) in the TME are very plastic, being able to switch their functions to inhibit or promote tumor progression in response to different environmental stimuli [36]. Activation of classical anti-tumor TAMs (M1 type) or polarization of pro-tumor TAMs (M2 type) into M1 TAMs are perceived to be inhibiting forces against various cancers [36, 37]. Recently, Yin et al. found that METTL3 in macrophages helps regulate tumor progression. Ablating macrophage METTL3 promoted tumor growth and lung metastasis [38]. It also reshaped the TME by enhancing infiltration of M1- and M2-like TAMs, upsetting their homeostasis, and recruiting regulatory T (Treg) cells into tumor sites [38]. METTL3 depletion in macrophages also reduced the efficacy of programmed cell death protein 1 (PD-1) blockade therapy, suggesting an immune-relevant function for macrophage METTL3 [38]. This finding was supported by the work from Tong et al. who found that METTL3-deficient macrophages produced subnormal levels of tumor necrosis factor (TNF)-α when stimulated with lipopolysaccharide (LPS) in vitro [39]. Ablating METTL3 in macrophages also increased susceptibility to bacterial infection and tumor growth [39]. When Dong et al. characterized TAMs by single-cell RNA sequencing (scRNA-seq), they found that C1q^+^ macrophages expressed a set of immunomodulatory ligands and that their functions were regulated by the METTL14. Also, METTL14 deficiency in macrophages inhibited the anti-tumor function of CD8^+^ T cells and promoted tumor growth [40]. In contrast to m^6^A writers’ positive roles in macrophages, knocking down the m^6^A reader YTHDF2 promotes macrophages to express LPS-induced inflammatory cytokines, such as IL-6, TNF-α, IL-1β, and IL-12, suggesting that YTHDF2 plays a negative regulatory role in LPS-induced inflammatory responses of macrophages [41]. Taken together, these findings highlight the complicated roles of m^6^A regulators in macrophages and suggest that METTL3/14 and YTHDF2 may be potential targets for cancer immunotherapy (Fig. 2B).

### Dendritic cells

Dendritic cells (DCs) are important antigen-presenting cells (APCs) that act as a bridge between innate and adaptive immune responses [42, 43]. DCs can either take up tumor antigens into the phagosome for presentation on major histocompatibility complex (MHC) class II ( MHC-II) molecules or transport the antigens to the cytosol for MHC class I ( MHC-I) presentation [44]. Further cross-presentation of antigens from DCs to CD8^+^ cytotoxic T lymphocytes (CTL) enables more accurate recognition and efficient killing of tumor cells by CD8^+^ T cells [45]. Recently, METTL3-mediated m^6^A modification was found to promote DC activation and maturation, causing them to present new antigens to and thereby activate T cells [46]. The underlying mechanism was the enhancement of the translation efficiency of CD40, CD80, and Toll/interleukin-1 receptor (TIR) domain-containing adaptor protein (TIRAP) by METTL3, leading to the secretion of proinflammatory cytokines [46]. METTL3-silenced DCs have reduced expression levels of MHC-II, costimulatory molecules (CD80, CD86), and inflammatory cytokines (IFN-γ, IL-12), thereby inducing immune tolerance and prolonging allograft survival in mouse heart transplantation [47]. This suggests that METTL3 helps maintain the mature properties of DCs. In contrast, Han et al. reported that the m^6^A reader YTHDF1 negatively regulates the anti-tumor immune responses of DCs [48]. Specifically, YTHDF1 enhanced the translation of mRNAs that encode lysosomal proteases, which can degrade antigens in lysosomes. Without YTHDF1, translation of lysosomal proteases was diminished, favoring antigen cross-presentation and promoting more CTL responses against tumors (Fig. 2C) [48].

### T cells

T cells are developed in the thymus, and when T cells mature, they migrate to peripheral organs and constitute the foundation of the adaptive immune system [49]. T cells offer important protection against viral infection and tumor cells [50]. They are generally classified into two groups depending on whether their cell surface receptor is CD4 or CD8 [51]. Depletion of METTL3 in CD4^+^ T cells disrupts T cell homeostasis and differentiation by downregulating the activation of IL-7-mediated STAT5/suppressor of cytokine signaling (SOCS) (Fig. 2D) [52]. Interestingly, depletion of METTL3 suppresses the function and stability of Treg cells by inhibiting IL-2/STAT5 signaling and promoting the cytokine secretion of T effector cells, resulting in enhancement of the anti-tumor immune responses in the TME (Fig. 2E) [53]. T follicular helper (Tfh) cells are a specialized CD4^+^ T cell subset critical to humoral immunity [54]. Yao et al. reported that conditional ablation of METTL3 in CD4^+^ T cells dampened Tfh differentiation and functional maturation, further inhibiting the antibody response of B cells by impairing the stability of m^6^A-modified *Tcf7* mRNA (Fig. 2D) [55]. A very recent study showed that ALKBH5 deficiency decreased the mRNA stability and protein secretion of IFN-γ and C-X-C motif chemokine ligand 2 (CXCL2) in CD4^+^ T cells, thereby alleviating experimental autoimmune encephalomyelitis (Fig. 2D) [56]. As IFN-γ is an important anti-tumor cytokine and ALKBH5 has a positive effect on the expression of IFN-γ in autoimmunity, ALKBH5 may have the potential to positively regulate the anti-tumor effect of CD4^+^ T cells. Therefore, the different functions of the m6A modification in T cells may depend on cell types and cellular contexts and this merits further investigation.

## Role of m^6^A modification in remodeling the tumor microenvironment

The TME plays an important role in cancer progression and significantly affects responsiveness to immunotherapy [57, 58]. In recent years, compelling evidence indicates that m^6^A regulators in tumor cells are associated with immune cell responses and immune checkpoint blockers (ICBs) efficacy. Alteration of the m^6^A modification in tumor cells influences the infiltration, activation, and effector functions of infiltrated immune cells in the TME [59], making targeting m^6^A—and especially m^6^A regulators in tumor cells—a promising strategy for improving cancer immunotherapy.

### m^6^A writers

METTL3 plays a dual role as either an oncogene or a tumor suppressor gene in various types of cancers, including hepatocellular carcinoma (HCC) [60, 61], hepatoblastoma [62], gastric cancer (GC) [63], colorectal cancer (CRC) [64], non-small cell lung cancer (NSCLC) [65, 66], and bladder cancer (BLC) [67, 68]. Abnormal expression of METTL3 in tumor cells affects the infiltration of immune cells. In testicular germ cell tumors (TGCT), METTL3 expression was significantly downregulated in TGCT tissues, and its level correlated positively with patient survival rates and levels of tumor-infiltrating CD8^+^ T cells, CD4^+^ T cells, and NK cells [69]. However, METTL3 was highly expressed in CRC tumor cells. Depletion of METTL3 or METTL14 in tumor cells increased cytotoxic tumor-infiltrating CD8^+^ T cells and elevated secretion of IFN-γ, CXCL9, and CXCL10 in the TME, thereby enhancing the response to anti-PD-1 treatment in mismatch-repair-proficient or microsatellite instability-low CRC tumors [70]. In cervical cancer (CC), METTL3 was expressed much more highly in tumor tissues than in tumor-adjacent tissues, and its level was positively related to the density of CD33^+^ myeloid-derived suppressor cells (MDSCs), which in turn was linked to poor patient survival rate [71]. Expression levels of METTL3 in the tumor also negatively correlated with breast cancer (BC) patient survival rate and tumor-infiltrating CD8^+^ T cells, helper T cells, and activated NK cells. In contrast, they positively correlated with M2 TAMs in BC [72]. For HCC, Shen et al. analyzed 433 samples from The Cancer Genome Atlas (TCGA) database, finding that METTL3 expression was negatively related to infiltration of DCs into tumors [73]. In head and neck squamous cell carcinoma (HNSCC), METTL3 and HNRNPC were highly expressed in tumor tissue than normal tissue, high expression of METTL3 and HNRNPC were positively associated with the infiltration of CD4 naive T cells, CD4 memory-activated T cells, and eosinophils [74].

As a critical component of the multicomponent methyltransferase complex, METTL14 is abnormally expressed and plays key regulatory roles in various cancers [75, 76]. In recent years, the effects of tumor cells’ METTL14 on the immune cells of the TME have only begun to be revealed. Low levels of METTL14 predicted an unfavorable prognosis in BC, in which METTL14 expression levels significantly and positively correlated with infiltrating levels of CD4^+^ T cells, CD8^+^ T cells, neutrophils, macrophages, and DCs. In contrast, they correlated negatively with Treg cells in BC [77]. In clear cell renal cell carcinoma (ccRCC), METTL14 possessed a good diagnostic and prognostic value. Also, METTL14 expression correlated negatively with Treg cells in BC, and the METTL14/CCL5/Tregs axis was a potential signaling pathway for regulating anti-tumor immunity in ccRCC [78].

In GC, WTAP was highly expressed in tumor cells, and its expression level correlated negatively with T cell infiltration and T cell-related immune responses, which was linked to poor prognosis [79]. In esophageal cancer (EC), however, high expression levels of WTAP were negatively associated with patient survival times and positively correlated with both immuno-inhibitors and immuno-stimulators such as cancer-associated fibroblasts, myeloid DCs, T cells, neutrophils, Treg cells, and macrophages (Table 1) [80].

**Table 1:** Role of m^6^A modifications in remodeling the tumor microenvironment

Regulators	Cancer type	Up- or down-regulated in tumor cells	Function	References
METTL3	TGCT	Down-regulated	Positively correlates with infiltration of CD8^+^ T cells, CD4^+^ T cells, and NK cells	[69]
METTL3	CRC	Up-regulated	Negatively correlates with infiltration of CD8^+^ T cells; Negatively correlates with IFN-γ, CXCL9, and CXCL10 secretion; Negatively correlates with the response to anti-PD-1 treatment	[70]
METTL3	CC	Up-regulated	Positively correlates with the density of CD33^+ ^MDSCs	[71]
METTL3	BC	Up-regulated	Negatively correlates with infiltration of CD8^+^ T cells, helper T cells, and activated NK cells; positively correlates with infiltration of M2 TAMs	[72]
METTL3	HCC	Up-regulated	Negatively correlates with infiltration of DCs	[73]
METTL3 HNRNPC	HNSCC	Up-regulated	Positively correlates with the infiltration of CD4 naive T cells, CD4 memory-activated T cells and eosinophils	[74]
METTL14	BC	Down-regulated	Positively correlates with infiltrating levels of CD4^+^ T cells, CD8^+^ T cells, neutrophils, macrophages, and DCs; negatively correlates with Treg cells	[77]
METTL14	ccRCC	Up-regulated	Negatively correlates with infiltration of Treg cells	[78]
WTAP	GC	Up-regulated	Negatively correlates with T cell infiltration and T cell-related immune responses	[79]
WTAP	EC	Up-regulated	Positively correlates with both the immuno-inhibitors and immuno-stimulators infiltration, such as myeloid DCs, T cells, neutrophils, Treg cells, and macrophages	[80]
FTO	AML	Up-regulated	Positively correlates with immune evasion, and inhibits the cytotoxicity of T cells	[85]
ALKBH5	Melanoma	unknown	Positively correlates with Treg cell infiltration; ALKBH5 deletion enhances the efficacy of anti–PD-1 therapy	[88]
ALKBH5	ICC	unknown	Positively regulates PD-L1 expression in tumor cells and inhibits the expansion and cytotoxicity of T cells by PD-1/PD-L1 signaling	[89]
YTHDF2	LGG	unknown	Positively correlates with infiltration of B cells, CD8^+^ T cells, CD4^+^ T cells, macrophages, neutrophils, and DCs	[92]
YTHDF1 YTHDF2	NSCLC	Up-regulated	Positively correlates with tumor-infiltrating lymphocytes, including CD8^+^ T cells, FOXP3^+^ T cells, PD-1^+^ T cells, and CD45RO^+^ immune cells	[93]
YTHDF2	KIRC	Down-regulated	Positively correlates with infiltration of B cells, CD8^+^ T cells, CD4^+^ T cells, macrophages, neutrophils, and DCs	[94]
YTHDF1	GC	Up-regulated	Negatively correlates with CD4^+^ cells and CD8^+^ T cells; positively correlates with MDSCs	[96]
YTHDF1	BC	Up-regulated	Negatively correlates with infiltration of CD4^+^ cells, CD8^+^ T cells, activated NK, and monocytes; positively correlates with infiltration of M1 macrophages	[97]

### m^6^A erasers

Upregulated expression of FTO intrinsically modulates genes in tumor cells that relate to malignant potential, promoting the progression of various types of cancer such as acute myeloid leukemia (AML) [81], glioblastoma (GBM) [82], CRC [83], and GC [84]. Genetic depletion or pharmacological inhibition of FTO dramatically attenuates leukemia stem/initiating cell self-renewal and reprograms immune responses by suppressing the expression of immune checkpoint genes such as leukocyte immunoglobulin-like receptor B4 (LILRB4) [85]. FTO inhibition sensitizes leukemia cells to T cell cytotoxicity and overcomes immune evasion induced by hypomethylating agents. As well as regulating immune checkpoint genes, FTO functions as an oncogene by reprogramming the glycolytic metabolism of tumor cells; it also further inhibits CD8^+^ T cell activity [86]. Targeting FTO with a small-molecule inhibitor blocks FTO-mediated immune evasion and synergizes with the PD-1/PD-L1 checkpoint blockade [86, 87]. Another eraser, ALKBH5, utilizes different mechanisms but performs a similar function in regulating anti-tumor responses in melanoma, and its expression in tumor cells correlates positively with Treg cell infiltration. ALKBH5 deletion enhances the efficacy of anti–PD-1 therapy in melanoma patients [88]. In addition, in intrahepatic cholangiocarcinoma (ICC), ALKBH5 positively regulates PD-L1 expression in tumor cells and inhibits the expansion and cytotoxicity of T cells through PD-1/PD-L1 signaling (Table 1) [89].

### m^6^A readers

Readers mediate the effects of the m^6^A modification by controlling the fate of modified RNA. Previous studies showed that upregulated expression of readers such as YTHDF1 promotes the progression of some cancers, such as NSCLC [90] and ovarian cancer [91]. The latest research also found that readers, mainly YTHDF1, YTHDF2, and YTHDC2, correlated positively with expression levels of several immune checkpoint receptors (including PD-1, T cell immunoglobulin and mucin-domain containing-3, and cytotoxic T-lymphocyte-associated antigen 4) as well as with the abundance of tumor-infiltrating lymphocytes (such as CD8^+^ T cells, CD4^+^ T cells, macrophages, and DCs) in several cancers, such as lower-grade glioma (LGG), NSCLC, KIRC and BC (Table 1) [92–99].

The above studies show that most of the m^6^A regulatory factors are abnormally expressed in tumors, where they create an immunosuppressive microenvironment. It would therefore seem beneficial to reshape the TME by enhancing or reducing m^6^A modification or by targeting m^6^A regulators to prevent that immunosuppression. However, current knowledge is still in its infancy. Further identification of other m^6^A-related modulators may greatly improve our understanding of tumor immune regulation and provide effective anti-tumor therapeutics for cancer patients.

## The potential role of the m^6^A modification in gut microbiota-mediated tumor immunity

Gut microbiota is a group of microorganisms that lives in the intestines of humans or other animals and is mutually beneficial and symbiotic with the body. Preclinical mouse models and clinical trials indicate that the gut microbiome modulates tumor response to ICBs [100–103]. Recent studies have revealed the interaction between host microbiota and RNA modifications. Transcriptome-wide profiling showed that the microbiome had a strong effect on host m^6^A modifications [104]. Lack of microbiome exposure resulted in higher m^6^A modifications in the brain and intestine in germ-free (GF) mice than specific pathogen-free (SPF) mice [104]. Another study reported that GF mice and SPF mice had differentially methylated peaks in the cecum, which were mainly associated with metabolic and inflammatory pathways [105]. Some specific microbial species, such as *Akkermansia muciniphila* and *Lactobacillus plantarum*, could directly change specific m^6^A modifications in mono-associated mice (animals colonized by only one microbial species) [105]. Currently, the relationship between m^6^A and gut microbiota in cancer development has not been explored. It is reported that virus infection, such as rotavirus and severe acute respiratory syndrome coronavirus 2 (SARS-CoV-2), induces global m^6^A modifications on mRNA transcripts of host cells and affects host immune defense against virus infections [106–109]. Therefore, a better understating of the potential functional interaction of gut microbes with the host m^6^A machinery may offer new strategies for the development of innovative therapies against cancer.

## Strategies and challenges of targeting m^6^A modification for cancer immunotherapy

In the above discussion, we concluded that m^6^A regulators, such as METTL3, YTHDF1, and YTHDF2, directly contribute to the regulation of anti-tumor immunity in different ways. Meanwhile, abnormal expression of m^6^A regulators in tumor cells leads to an immunosuppressive TME, further accelerating cancer progression. Therefore, targeting m^6^A modification should 1) directly inhibit tumor cell growth, 2) enhance the anti-tumor ability of immune cells, as by boosting the cytotoxicity of CD8^+^ T cells and NK cells, 3) remodel the TME by reprogramming M2 TAMs into M1 TAMs. Here, we discuss strategies and challenges inherent in targeting m^6^A modification for cancer immunotherapy.

### Developing specific inhibitors against m^6^A regulators to boost endogenous anti-tumor immunity

**Fig. 3:**
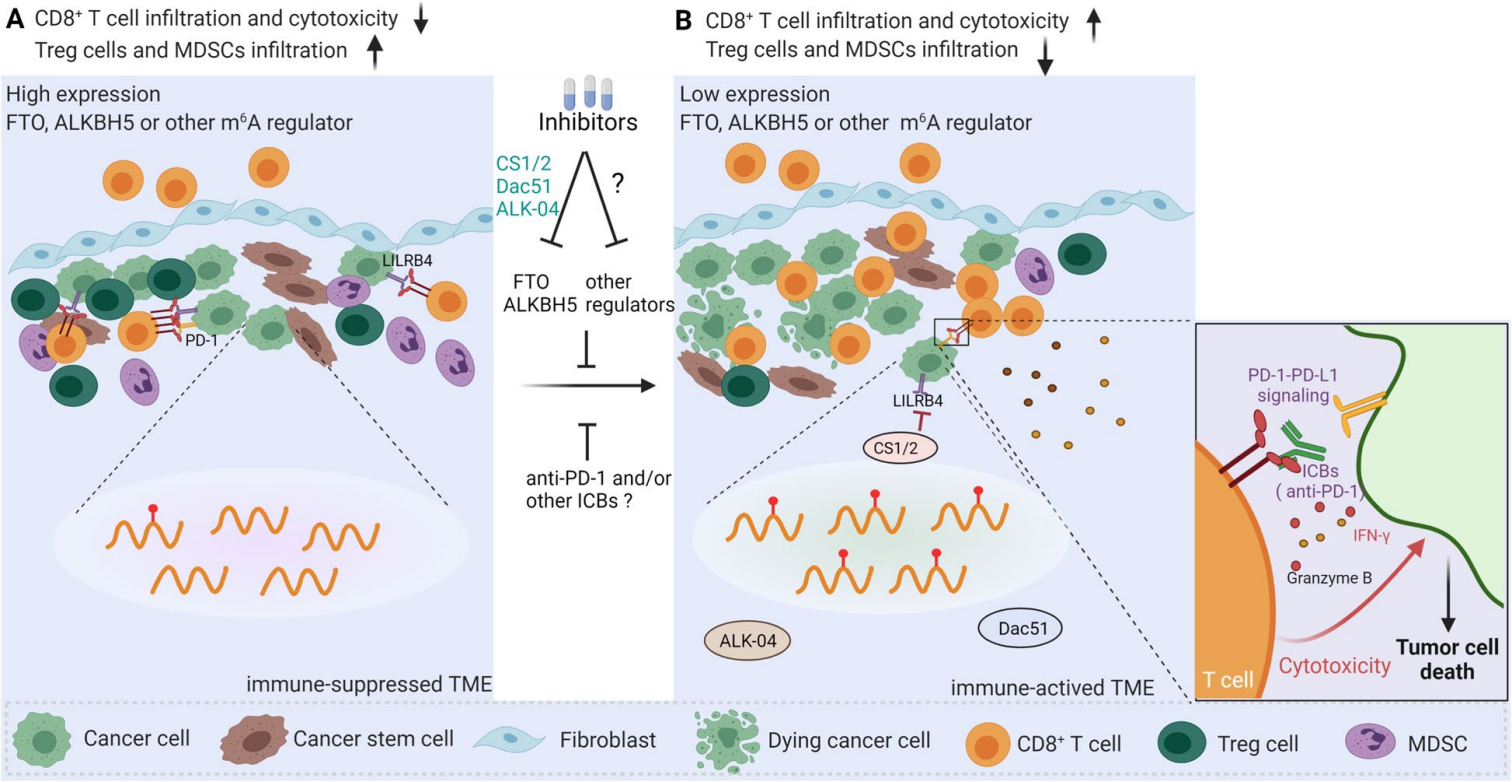
Inhibitors of m^6^A regulators in tumor cells indirectly augment T cell trafficking and decrease immunosuppression. **A** High expression in tumor cells of m^6^A regulators, such as FTO, ALKBH5, and others, leads to an immune-suppressed TME characterized by high expression of immune checkpoints [PD-1 and leukocyte immunoglobulin-like receptor B4 (LILRB4)], reduced infiltration, decreased cytotoxic function of CD8^+^ T cells, and enhanced infiltration of Treg cells and myeloid-derived suppressor cells (MDSCs). **B** Targeting FTO or ALKBH5 with specific inhibitors, such as CS1/2, Dac51, or ALK-04, or combining with ICBs, reverses the immunosuppressive TME by increasing the infiltration and cytotoxicity of CD8^+^ T cells and inhibiting the infiltration of Treg cells and MDSCs, thereby creating an immune-activated TME. Figure created with BioRender.com

It is recognized that most of the m^6^A regulators are abnormally high-expressed in tumor cells. Therefore, targeted inhibition of those regulators should be an effective way to directly inhibit tumor cell proliferation and further activate endogenous anti-tumor responses that kill cancer cells or cancer stem cells. In recent years, a series of small-molecule inhibitors targeting m^6^A regulators such as FTO, ALKBH5, and METTL3 have been developed, but among them, FTO is the most attractive target. Between 2012 and 2019, researchers developed and identified a set of FTO inhibitors, such as rhein, MO-I-500, meclofenamic acid (MA), fluorescein, 2-hydroxylglutarate (R-2HG), FB23, and FB23-2, that showed marked anti-tumor effects in vitro and in vivo (Table 2) [110–115]. Between 2020 and the present, several upgraded FTO inhibitors have been developed, including CS1/CS2 [85] and Dac51 [86], which not only suppress cancer cell proliferation and cancer stem cell self-renewal but also improve anti-tumor immunity (Fig. 3). Most importantly, they promote the infiltration and cytotoxicity of CD8^+^ T cells [85, 86].

**Table 2:** Specific inhibitors against m^6^A regulators

Targets	Inhibitors	Function	References
FTO	rhein	Inhibits FTO activity on m^6^A demethylation	[110–113]
	MO-I-500		
	meclofenamic acid		
	fluorescein		
	R-2HG	Anti-leukemia and anti-glioma	[114]
	FB23/FB23-2	Inhibits the proliferation and promotes cell differentiation/apoptosis of human acute myeloid leukemia cells	[115]
	CS1/CS2	Inhibits cancer cell proliferation, cancer stem cell self-renewal and immune evasion	[85]
	Dac51	Promotes T cell response and enhances the anti-PD-1 therapy	[86]
ALKBH5	2-{[1-hydroxy-2-oxo-2-phenylethyl] sulfanyl} acetic acid, 4-{[furan-2- yl]methyl}amino-1,2-diazinane-3,6- dione	Suppresses the proliferation of leukemia cell lines (HL-60, CCRF-CEM, and K562)	[116]
	ALK-04	Inhibits the infiltration of Treg cells and MDSCs, enhances the anti-PD-1 therapy	[88]
METTL3/ME TTL14	STM2457	Prevents AML expansion and reduces the number of leukemia stem cells in vivo	[117]

With continuous technological breakthroughs, screening of ALKBH5 inhibitors is also proceeding steadily. Two ALKBH5 inhibitors—2-{[1-hydroxy-2-oxo-2-phenylethyl]sulfanyl} acetic acid and 4-{[furan-2-yl]methyl}amino-1,2-diazinane-3,6-dione—suppress the proliferation of leukemia cell lines (HL-60, CCRF-CEM, and K562) when used in micromolar amounts (Table 2) [116]. In 2020, Li et al. identified a specific inhibitor of ALKBH5, named ALK-04, which decreases the infiltration of Treg cells and MDSCs and inhibits tumor growth by enhancing the efficacy of anti-PD-1 therapy (Fig. 3) [88]. Recently, through high-throughput screening of 250,000 drug-like compounds, Eliza et al. discovered a highly potent and selective first-in-class inhibitor of METTL3 and METTL14, named STM2457 (Table 2). This inhibitor showed significant antileukemic effects in preclinical AML models, providing proof of concept that targeting m^6^A writers is a promising strategy for cancer therapy [117].

Although several inhibitors that target m^6^A regulators have been developed, there are still the following challenges: 1) Inhibitors that target readers, which decode the m^6^A mark to mediate downstream effects of m^6^A-modified mRNAs, have not been discovered. The possible reason is that the binding sites of a reader might be overlapping with other readers with opposed functions, which could have both beneficial and detrimental effects on cancer growth. A recent screening of clustered regularly interspaced short palindromic repeats (CRISPR)/CRISPR-associated protein 9 (Cas9) identified YTHDF2 as a therapeutic target for triple-negative breast cancer [118]. In this study, the authors used an alternative approach to inhibit YTHDF2’s effects by targeting its downstream effectors to directly stabilize the target mRNAs or overexpress the proteins that they encode. 2) The established m^6^A small-molecule inhibitors are being used mainly in hematological malignancies. The main reason is that the role and associated mechanism of the m^6^A modification in solid tumors still have not yet been fully understood. It is worth noting that m^6^A plays either tumorigenesis or tumor suppressor roles in the development of solid tumors, such as glioblastoma [119, 120] and colorectal cancer [121, 122], while in general speaking, the m^6^A modification plays a promoting role in hematological malignancies. Another reason is the complexity of the TME of solid tumors, which is tightly associated with the clinical outcome of cancer patients [123]. In a couple of solid tumors, the other group and we have uncovered beneficial roles for regulators, such as METTL3 and YTHDF2, in the immune response to tumor cells by NK cells [33, 34]. Therefore, m^6^A inhibitors that target tumor cells may impair the anti-tumor immune response. A comprehensive understanding of the different roles of m^6^A in regulating immune cells and tumor cells within the TME is needed to develop m^6^A-based targeted drugs with the specificity to distinguish tumor cells from immune cells during immunotherapy. 3) Preventing the exhaustion of anti-tumor immune cells and activating endogenous anti-tumor immunity are two effective approaches to treat tumors. However, most current inhibitors affect mainly tumor cells, though a few, such as CS1/2, Dac51, and ALK-04, can activate endogenous anti-tumor immune cells or decrease the infiltration of immunosuppressive cells. 4) Most inhibitors are still at the preclinical stage, and therefore, their therapeutic value needs to be explored in the clinic. Some pharmaceutical or biotech companies (e.g., STORM Therapeutics, Accent Therapeutics, Gotham Therapeutics, and Genovel Biotech Corp.) have started to develop highly potent and selective small-molecule inhibitors that directly target m^6^A regulators such as METTL3, METTL14, FTO, and ALKBH5 [30]. In addition, PROTAC (proteolysis targeting chimera)-based or molecular glue-based inhibitors could be developed to selectively degrade dysregulated m^6^A regulatory proteins for cancer therapy [30, 124]. Also, gene silencing using oligonucleotide-based therapeutics (ONTs), such as small interfering RNA (siRNA), antisense oligonucleotides, or decoy oligodeoxynucleotides, is a promising strategy for targeting m^6^A regulators in tumor cells and immune cells.

### Developing an effective programmable m^6^A editing system

Most investigations of m^6^A change overall RNA methylation by interfering with m^6^A regulators, or they explore regional functions of m^6^A by mutating methylation sites [125]. However, mutating a methylation site changes mRNA nucleotide sequence and introduces other unknown features, complicating the interpretation of the phenotype. Moreover, the distribution of m^6^A on mRNA is not uniform, as most of these modifications cluster near the stop codon [126]. Recently, several groups have attempted to manipulate m^6^A in a site-specific manner by using CRISPR/Cas9 technology, which enables precise genome editing, including targeted DNA cutting/repair, direct base editing, and site-specific epigenome editing [127, 128]. In 2019, Liu et al. created the first programmable RNA m^6^A editing machinery by fusing CRISPR-dead Cas9 (dCas9), which lacks the ability to cut DNA, with a single-chain m^6^A methyltransferase or demethylase [129]. These engineered m^6^A writers or erasers can install or remove m^6^A at specific sites without changing the primary sequence. Subsequently, other groups have developed and optimized precise m^6^A editors by replacing dCas9 with smaller dCas13 [130–132]. This new platform enables efficient manipulation of individual m^6^A sites within endo-transcripts with minimal off-target alterations in both normal mammalian cells and cancer cells. Thus, m^6^A editors offer a powerful approach to fine-tuning the mRNA modification of specific genes and their related biological functions. Further investigations are warranted to develop smaller m^6^A editors that allowed for easier cell delivery with higher specificity and more adjustable features for immunotherapy that targets the m^6^A modification.

### Developing an effective m^6^A modification system of adoptive cell therapy

NK cells account for 10%–20% of human peripheral blood leukocytes, and they are an attractive source of immunotherapy based on genetically modified immune cells [31, 133, 134]. Adoptive NK cell-based immunotherapy, such as chimeric antigen receptor (CAR) NK cells and induced pluripotent stem cell (iPSC)-derived NK or CAR NK cells, are effective for anti-tumor therapy [31, 135–137]. However, it seems to be difficult to obtain a large number of NK cell*s* in a short time in vitro due to the limited supply of initial primary or initial iPSC-derived NK cells [138, 139]. Current studies have found that adding cytokines (IL-2 + IL-15) or co-cultivating with K562 feeder cells greatly improves the expansion efficiency of NK cells in vitro [136, 137, 140]. As cell culture time increases, however, the proliferation efficiency and effector functions of NK cells may decrease. Therefore, increasing the expression of genes that promote the proliferation of NK cells and enhance their effector functions may be an effective way to increase the efficiency of NK expansion in vitro.

**Fig. 4:**
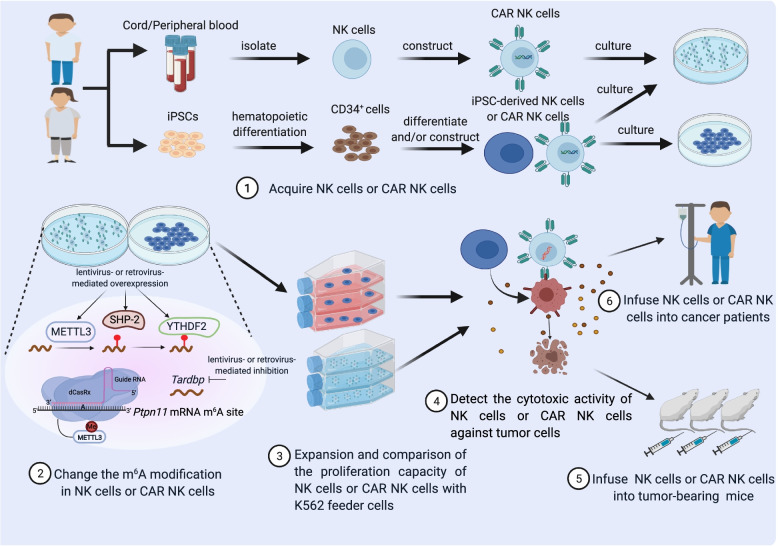
m^6^A modification strategies for NK cell-based immunotherapy. During the production of CAR NK and iPSC-derived NK cells, several approaches targeting the m^6^A modification can be used to increase expansion in vitro. They include lentivirus- or retrovirus-mediated gene delivery of METTL3, YTHDF2, and SHP-2; short hairpin RNA interference targeting *Tardbp*; and m^6^A editing machinery that manipulates the m^6^A site in *Ptpn11* mRNA. These m^6^A-based strategies may improve the functionality and proliferation of NK cells. Figure created with BioRender.com

Our lab and another group found that in mice the m^6^A regulators METTL3 and YTHDF2 and their corresponding target genes, *Ptpn11* (encoding SHP-2)*,* and *Tardbp* (encoding TDP-43), associated with a gain of effector function and proliferation in NK cells [33, 34]. With lentivirus- or retrovirus-mediated overexpression of METTL3, SHP-2, or YTHDF2, knockdown of *Tardbp,* or dCasRx-conjugated METTL3 to manipulate methylation events at target mRNA m^6^A sites of *Ptpn11*, it might be possible to improve the effector function and proliferation ability of NK cells (Fig. 4). As far as we know, there have been no attempts to modulate m^6^A regulators to enhance the proliferation and cytotoxicity of human NK cells in vitro. As the m^6^A regulatory site is highly conserved between humans and mice [18], such investigation might help inform future protocols for the optimal generation of more CAR NK cells and iPSC-derived NK cells.

Besides CAR NK cells and iPSC-derived NK cells therapy, CAR T cell therapy is one of the most successful cancer immunotherapies in the clinic [6]. As discussed above, m^6^A regulators, such as ALKBH5 and METTL3, and their targeting genes affect T cell function (Fig. 2D). Therefore, modulating m^6^A modifications in CAR T cells may also represent a potentially promising strategy to enhance anti-tumor immune responses.

### Encapsulating m^6^A modification molecules into nanoparticles to specifically target TAMs

Nanoparticles (NPs) platforms have emerged as promising carriers in cancer therapy [141]. In recent years, scientists have developed NPs that can precisely and efficiently deliver mRNAs, siRNAs, and protein-based drugs into tumor cells [142, 143]. Recent research in HCC found that METTL3 can stabilize the RNA transcript of a long non-coding RNA-LINC00958 via m^6^A modification, and aberrant overexpression of LINC00958 is an important cause of accelerated HCC. Moreover, specifically delivering NP-encapsulated siRNA of LINC00958 to tumor cells in the TME reduced m^6^A modification in LINC00958 and inhibited the progression of HCC [144].

**Fig. 5:**
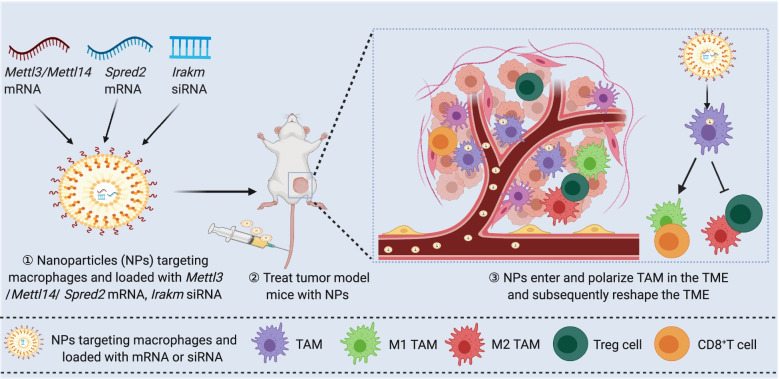
Nanoparticles (NPs) encapsulating m^6^A modification molecules can specifically target TAMs. NPs that deliver *Mettl3, Mettl14*, or *Sp red2* mRNA or *Irakm* siRNA specifically into TAMs can reprogram the macrophages from the M2-type to the M1-type. This switch reshapes the TME and inhibits tumor progression. Figure created with BioRender.com

Similar to being used for targeted delivery of drugs into tumors cells, many kinds of NPs, such as lipid-based NPs [145], polymer-based NPs [146], and inorganic NPs [ 147], can be recognized by TAMs and then deliver drugs or RNA into TAMs of the TME [148]. For example, C-C motif chemokine receptor 2 siRNA-loaded lipid NPs prevent the recruitment of TAMs into the tumors [145]. Polymeric NPs can be loaded with siRNAs to target vascular endothelial growth factor and placental growth factor signaling in both tumor cells and M2 TAMs, skewing the immunosuppressive M2 TAMs to the M1 type and thereby inhibiting tumor growth [146]. Recent studies found that METTL14, METTL3, and their target genes, *Spred2* and *Irakm,* in macrophages are associated with tumor progression [38, 39]. Therefore, targeted delivery of NP-encapsul ated *M ettl3 *, *Mettl14*, or *Spred2* mRNA or of *Irakm* siRNA into TAMs might promote TAM polarization, reduce Treg cell infiltration, promote the cytotoxic function of CD8^+^ T cells, and reverse immunosuppression in the TME (Fig. 5).

## Conclusions and perspectives

Although the biological sequelae of m^6^A modification in tumors have been extensively investigated in recent y ears, strategies for developing m^6^A-based targeted drugs for cancer immunotherapy are still in their infancy. In this review, we summarized current progress in understanding the roles and mechanisms of m^6^A regulators in immune cells and their effects on immune responses in the TME. It is important to acknowledge that the ways in which the RNA m^6^A machinery affects immune cells and tumor cells are complicated and still not well understood. For example, METTL3 regulates DC functions positively, whereas YTHDF1 regulates them negatively. Similar discrepancies are found in macrophages, where METTL3/14 exert positive control on polarization and immune functions, whereas YTHDF2 exerts negative control. Moreover, both METTL3 and YTHDF2 are positive regulators of NK cell effector functions. Thus, additional studies of the roles of other potential regulators, such as YTHDF3, FTO, and ALKBH5, in immune cells are warranted. In addition, as most m^6^A regulators play oncogenic roles in tumor development, m^6^A-based inhibitors that target tumor cells may have opposite effects to those intended as inhibition of m^6^A and impair host anti-tumor immune responses. Therefore, a more comprehensive understanding of how m^6^A modulators regulate immune cells and tumor cells will expand our knowledge of the biological roles of m^6^A in anti-tumor immunity and help with the development of m^6^A-based targeted drugs with the specificity to distinguish tumor cells from immune cells during immunotherapy.

We also proposed four reasonable strategies for improving m^6^A-based cancer immunotherapy, including developing: 1) inhibitors against m^6^A regulators to modulate anti-tumor immunity, 2) a programmable m^6^A gene-editing system to manipulate individual m^6^A sites, 3) m^6^A-modified system for adoptive cell therapy, 4) nanoparticles that can specifically deliver m^6^A modification molecules into TAMs to remodel the TME. Of note, ICBs therapy has achieved remarkable success in the treatment of cancer. As discussed above, m^6^A modifications not only modify the immune checkpoint expression pattern in many types of cancers [89, 92–99] but also regulate the sensitivity and efficacy of ICBs therapy in several preclinical animal models [38, 86–89]. Although there are no clinical trials using m^6^A inhibitors for the treatment of cancer yet, it is reasonable to speculate a synergistic anticancer effect through the combination of m^6^A inhibitors and ICBs. Future clinical trials are needed to prove this in humans.

Overall, m^6^A modification is dynamic and reversible, and many of its regulators are yet to be discovered. Targeting m^6^A modification for cancer immunotherapy is challenging because the expected “on-target, off-tumor” activity may potentially lead to significant toxicity. Therefore, a better understanding of this process and the ability to control it accurately are still significant challenges that warrant further study.

## Data Availability

Not applicable.

## Abbreviations

m^6^A: *N*^6^-Methylation of adenosine
TME: Tumor microenvironment
TAMs: Tumor-associated macrophages
rRNA: Ribosomal RNA
tRNA: Transfer RNA
m^1^A: *N*^1^-Methyladenosine
m^5^C: 5-Methylcytosine
m^7^G: *N*^7^-Methylguanosine
CAR: Chimeric antigen receptor
METTL3: Methyltransferase-like 3
METTL14: Methyltransferase-like 14
RBM15: RNA binding motif protein 15
WTAP: Wlms tumor 1 associated protein
VIRMA: Vir-like m^6^A methyltransferase associated subunits
ZC3H13: Zinc finger CCCH domain-containing protein 13
UTRs: Untranslated regions
FTO: Fat mass and obesity-associated protein
ALKBH5: Alpha-ketoglutarate-dependent dioxygenase alkB homologue 5
YTHDC1: YTH domain containing 1
YTHDC2: YTH domain containing 2
HNRNP: Heterogeneous nuclear ribonuclease
FMRP: Fragile x mental retardation protein
YTHDF1: YTH domain family 1
YTHDF2: YTH domain family 2
YTHDF3: YTH domain family 3
IGF2BPs: Insulin-like factor-2 mRNA binding proteins
PRRC2A: Proline rich coiled-coil 2A
NK cells: Natural killer cells
IL-15: Interleukin 15
STAT5: Signal transducer and activator of transcription 5
Eomes: Eomesodermin
SHP-2: SH2 domain-containing protein tyrosine phosphatase-2
Treg cells: Regulatory T cells
PD-1: Programmed cell death protein 1
LPS: Lipopolysaccharide
scRNA-seq: Single-cell RNA sequencing
DCs: Dendritic cells
APCs: Antigen-presenting cells
MHC-II: MHC class II
MHC-I: MHC class I
CTL: Cytotoxic T lymphocytes
TIRAP: Toll/interleukin-1 receptor domain-containing adaptor protein
Tfh cells: T follicular helper cells
CXCL2: C-X-C motif chemokine ligand 2
ICBs: Immune checkpoint blockers
HCC: Hepatocellular carcinoma
GC: Gastric cancer
CRC: Colorectal cancer
NSCLC: Non-small cell lung cancer
BLC: Bladder cancer
TGCT: Testicular germ cell tumors
CC: Cervical cancer
MDSCs: Myeloid-derived suppressor cells
BC: Breast cancer
TCGA: The Cancer Genome Atlas
HNSCC: Head and neck squamous cell carcinoma
ccRCC: Clear cell renal cell carcinoma
EC: Esophageal cancer
AML: Acute myeloid leukemia
GBM: Glioblastoma
LILRB4: Leukocyte immunoglobulin like receptor B4
ICC: Intrahepatic cholangiocarcinoma
LGG: Lower-grade glioma
GF: Germ-free
SPF: Pathogen-free
SARS-CoV-2: Severe acute respiratory syndrome coronavirus 2
MA: Meclofenamic acid
R-2HG: 2-Hydroxylglutarate
CRISPR: Clustered regularly interspaced short palindromic repeats
Cas9: CRISPR-associated protein 9
PROTAC: Proteolysis targeting chimera
ONTs: Oligonucleotide-based therapeutics
siRNA: Small interfering RNA
dCas9: CRISPR-dead Cas9
iPSC: Induced pluripotent stem cell
NPs: Nanoparticles
